# Ultrasound, laboratory and histopathological insights in diagnosing papillary thyroid carcinoma in a paediatric population: a single centre follow-up study between 2000-2022

**DOI:** 10.3389/fendo.2023.1170971

**Published:** 2023-05-18

**Authors:** Dominika Januś, Małgorzata Wójcik, Anna Taczanowska-Niemczuk, Aleksandra Kiszka-Wiłkojć, Monika Kujdowicz, Małgorzata Czogała, Wojciech Górecki, Jerzy B. Starzyk

**Affiliations:** ^1^ Department of Pediatric and Adolescent Endocrinology, Chair of Pediatrics, Institute of Pediatrics, Jagiellonian University, Medical College, Krakow, Poland; ^2^ Department of Pediatric and Adolescent Endocrinology, University Children’s Hospital, Krakow, Poland; ^3^ Department of Pediatric Surgery, Institute of Pediatrics, Jagiellonian University, Medical College, Krakow, Poland; ^4^ Department of Pediatric Surgery, University Children’s Hospital, Krakow, Poland; ^5^ Department of Pathology, University Children’s Hospital, Krakow, Poland; ^6^ Department of Pathomorphology, Jagiellonian University, Medical College, Krakow, Poland; ^7^ Department of Pediatric Oncology and Hematology, Institute of Pediatrics, Jagiellonian University, Medical College, Krakow, Poland; ^8^ Department of Pediatric Oncology and Hematology, University Children’s Hospital, Krakow, Poland

**Keywords:** autoimmune thyroiditis, papillary thyroid carcinoma, ultrasonography of thyroid gland, PTC subtypes, prepubertal PTC, pubertal PTC, adolescent PTC

## Abstract

**Background:**

Papillary thyroid carcinoma (PTC) often coincides with autoimmune thyroiditis (AIT); whether this association is incidental or causal remains debated.

**Objective:**

To evaluate the ultrasonographic, laboratory, and histopathological features of PTC in paediatric patients with and without AIT and its relationship to puberty.

**Design:**

A retrospective cohort study.

**Patients and methods:**

A retrospective analysis of medical records of 90 patients (69; 76.7% females). The mean age at PTC diagnosis was 13.8 years [range 6-18]. All patients were evaluated ultrasonographically before thyroid surgery. Thyroid nodules were categorised using the European Thyroid Imaging Reporting and Data System (EU-TIRADS PL), and cytopathology was assessed using Bethesda criteria. Neck ultrasound results and thyroid and autoimmune status were correlated with histopathological PTC assessment.

**Results:**

The coexistence of PTC and AIT was found in 48.9% (44/90) of patients. The percentage of AIT was increasing with age; AIT was present only in 1/3 of prepubertal, close to 50% in pubertal, and over 60% in adolescent patients. The youngest patients (aged <10 years old) presented more often with goitre and lymphadenopathy and less often with AIT than adolescents (15-18 years of age). There were no differences in TPOAb, TgAb, and TSH levels between the age subgroups. Presurgical TgAb levels were higher than those of TPOAb in the youngest patients. Histopathological analysis revealed that the solid subtype was observed more often in prepubertal children and diffuse sclerosing in children below 14 years of age, whereas the classic subtype dominated in late pubertal. Univariate and multivariate analyses revealed that lymph nodes metastases (LNM) were associated with PTC diameter and fT4 level, whereas extrathyroidal extension with age and angioinvasion with PTC diameter and age. The correlations between age and fibrosis, and the presence of psammoma bodies in malignant tissues were close to significant. We did not observe an association between TSH levels and the presence of autoimmunity and PTC variables.

**Conclusions:**

In paediatric patients the natural course of PTC may be less aggressive in adolescent patients than in younger children (especially < 10 years of age). We suggest that pre-operative evaluation of paediatric patients with thyroid nodules could include apart from assessment of thyroid hormones, evaluation of TPOAb, TgAb, and TRAb together with comprehensive neck ultrasonography.

## Introduction

The most common aetiology of acquired thyroid disease is autoimmune thyroiditis (AIT), reported in ~5-6% of paediatric patients, (0.3-2% of children and 4-9.6% of adolescents) (1–3). Thyroid carcinoma accounts for 12% of cancers, and in terms of incidence ranks seventh in children below 14 years, and fourth in adolescents (15-18 years) (4). The first report on the coincidence of thyroid cancer and AIT was presented by Dailey et al. in 1955, which suggested that AIT might be considered a precancerous lesion, as chronic inflammation contributes to the development of cancer in many tissues (5–8). Cell-mediated autoimmune inflammation of the thyroid may cause functional and/or structural disorders (9).

In adults with nodular AIT, the incidence, extent, and multifocality of papillary thyroid carcinoma (PTC) are increased (10–14). The correlation between AIT and PTC in paediatric patients is still under discussion. Thyroid nodules are more common in children with AIT (3.5%–31.5%) than in those without (0.5–2%) (15–22). In children with thyroid nodules, the risk of PTC is estimated to be 19-39%, and in nodular AIT, the risk of PTC ranges from 5% to 25%; however, the incidence of PTC in AIT cohorts is lower (0.67% to 9.6%) (9, 15–18, 23–25). A higher association between AIT and PTC has been reported in surgical series, ranging between 6.3-43% in paediatric patients with PTC (16, 26–32). Based on a review of reports published in 2000–2020, Sur et al. concluded that the development of PTC in children with AIT appeared to be higher than that in the healthy population (33). In contrast, Radetti et al. found that AIT contributes to the development of thyroid nodules but not cancer in children and adolescents (15). Similar observations have been reported by Ben-Skowronek et al. (34) and Borysewicz-Sanczyk et al. (35). These discrepancies might be related to how AIT was verified, whether by presurgical confirmation of autoimmunity in blood tests and typical ultrasound images, or by those two variables combined with histopathological assessment after thyroidectomy.

The clinical, molecular, and pathological presentations of paediatric PTC differ from those of adult PTC (36, 37). From a histopathological standpoint, paediatric PTC presents more often as a lesion in the thyroid tissue, rather than a single encapsulated nodule seen more frequently in adult PTC (37). Paediatric PTC is also characterized by large lesions, multifocality, bilaterality, and multiple satellite malignant lesions spread unilaterally or bilaterally, extrathyroidal extension (ETE) beyond the thyroid capsule, neck lymph node metastases (in over 80% of patients, even if the main malignant lesion is < 1cm), and lung metastases (present in 10–20% of the cases) (36). This severe presentation, particularly in children below 10 years of age, may be related to late diagnosis or an aggressive course (38). Despite this presentation, paediatric PTC has an excellent prognosis; the five-year survival is > 99% (4, 39–44).Treatment consists of thyroidectomy with radioactive iodide, used to treat metastatic or high-risk tumours (36). The most common genetic alterations in paediatric PTC are *RET-PTC* and *NTRK* fusions, whereas mutations in *BRAF, V600E*, and *RAS* are less frequent than in adults (45, 46).

Nonetheless, the paediatric population is not homogeneous, considering the influence of puberty (36, 37, 45, 47, 48). Few studies have compared differences in the biology of paediatric PTC between age groups. In two large paediatric studies, the authors reported significant differences between early childhood and adolescent PTC but did not relate the differences to thyroid autoimmunity (47, 48).

Although PTC is a rare cancer in children, its prevalence has increased in recent years. One of the causes is more frequent ultrasound (US) assessments and active ultrasound surveillance of thyroid disorders; however, many new patients present with advanced disease (4, 39, 40). Therefore, because of the potential link between AIT and PTC, careful follow-up of patients with AIT is necessary, and Polish recommendations state that children with AIT should undergo thyroid US screening at least once a year (49). In AIT, the ultrasound appearance of the thyroid gland varies depending on the phase and severity of the chronic inflammation (18, 50). Typical sonographic features include diffuse heterogeneity and nodular structures (18, 50). The ultrasound features of thyroid nodules suggestive of malignancy are solid composition (usually hypoechoic), irregular shape and margins, taller-than-wide shape, presence of microcalcifications, intranodular vascularity greater than peripheral vascularity, and cervical lymph node enlargement (51). In children and adolescents, any focal lesion found on US should be subjected to ultrasound-based malignancy risk stratification (36, 51). In this study, we used the EU-TIRADS-PL score, which is a modified EU-TIRADS score (51, 52).

The aim of the present study was to evaluate the ultrasonographic, laboratory, and histopathological variables of PTC in paediatric patients with and without AIT, and in relation to puberty.

## Subjects and methods

### Subjects

258 pediatric patients were referred for thyroid surgery between 2000 and 2022 in the major tertiary pediatric center in South-Eastern Poland (University Children’s Hospital in Krakow). Ninety patients out of this group (34.9%), all with confirmed PTC, with a mean age of 13.8 years (age range 6-18 years, 69 [76.7%] girls) were further evaluated and presented in the study.

### Methods

The retrospective analysis of medical records included the evaluation of thyroid function status and ultrasound, cytological, and histopathological variables in patients with PTC. All hormonal and immune assessments were routinely performed at the Department of Biochemistry at the University Children’s Hospital in Krakow, Poland and were determined in a single fasting blood sample. TSH, fT3, and fT4 levels were measured using immunochemistry with an ADVIA Centaur machine, and TPOAb, TgAb, and TRAb levels were assessed using a radioimmunoassay method with a Brams machine. All assessments were performed before inclusion in the study and prior to therapy with levothyroxine or antithyroid thiamazole, when needed. Molecular analyses were routinely performed for suspected genetic syndromes.

AIT was diagnosed based on typical features of chronic autoimmune thyroiditis on thyroid ultrasound assessment, as described previously, and increased TPOAb and/or TgAb and/or TRAb antibody levels, as well as after histopathological confirmation in all patients after thyroidectomy (18).

Thyroid ultrasound was performed for all patients at the time of thyroid dysfunction diagnosis. At our institution, thyroid ultrasound is routinely performed as active surveillance once a year in patients with thyroid dysfunction and as a screening in all patients newly referred for endocrine assessment. Ultrasonography (US) of the thyroid gland was performed at the University Children’s Hospital by paediatric endocrinologists and surgeons with experience in paediatric US (DJ, AT > 20 years and AKW, MW > 15 years). Thyroid US was performed using a high-resolution Voluson 730 GE Medical System (8–12-MHz linear-array transducer), Philips Epiq5 (L12-5 linear transducer), Philips iE22 (L11-3 linear transducer), and Samsung HS40 with elastography (LA3-16AD transducer). US was performed in axial and longitudinal planes. The analysis included ultrasound features of the thyroid gland according to the EU-TIRADS PL 2022 classification (Polish update of EU-TIRADS 2017) (51, 52), lymph nodes, and elastography.

Fine needle aspiration biopsy (FNAB) results were classified according to Bethesda criteria (53). Patients underwent total thyroidectomy with central and, if necessary, lateral lymph nodes dissection. Histopathological evaluation was performed at the Department of Pathology of University Children’s Hospital in Krakow. Postoperative staging was performed based on the tumour, nodes and metastases (TNM) system proposed by the American Joint Committee on Cancer (54).

This study was approved by the relevant institutional review board (The Bioethics Committee of the Jagiellonian University opinion number:1072.6120.288.2021). Written informed consent was obtained from all participants and/or their parents. Written informed consent was obtained from the individual(s) and minor(s) legal guardian/next of kin for the publication of any potentially identifiable images or data included in this article.

### Statistics

The baseline and demographic characteristics were summarised using descriptive statistics. Categorical variables are expressed as percentages. We assessed the distribution of continuous variables using the Shapiro–Wilk test and described continuous variables using median or mean and standard deviation as appropriate. The sensitivity, specificity, and positive and negative predictive values of ultrasonography of thyroid and lymph nodes were calculated in relation to the histological results. We assessed the absence of hyperechoic hilum, echogenicity of nodules, microcalcifications, and increased chaotic vascularisation in lymph nodes (combined). We used the non-parametric tests to compare groups PTC (+) AIT+/- and LNM+/- with PTC variables. We used the non-parametric tests to compare PTC(+) AIT+/- and LNM+/- groups with control groups [PTC(-) AIT(+)]. We used the Spearman test to search for correlations between clinical parameters and histological variables. A two-tailed p-value of <0.05 was considered statistically significant. The association of subject characteristics (female sex, age, familial history, AIT, TSH, fT4) and tumour diameter with cancer characteristics (fibrosis, angioinvasion, lymph node infiltration, multifocality, ETE, psammoma bodies) was evaluated using univariate and multivariate logistic regression. Characteristics with p<0.1 in univariate analysis were included in the multivariate logistic regression as p<0.1 is considered as a trend, p<0.05 as significant. All analyses were performed using STATISTICA version 13 (TIBCO Software Inc., Palo Alto, CA 94304 United States).

## Results

### Patients characteristics

258 pediatric patients were referred for thyroid surgery between 2000 and 2022 in the major tertiary pediatric center in South-Eastern Poland (University Children’s Hospital in Krakow). Histopathological diagnoses were updated according to the 2022 WHO Classification of Thyroid Tumours (37). Eighty-four patients had thyroid follicular nodular disease, 21 had Graves disease unresponsive to therapy, two had non-invasive follicular thyroid neoplasm with papillary-like nuclear features (NIFTP), two had well-differentiated tumour of uncertain malignant potential (WDUMP), 11 had follicular tumour of uncertain malignant potential (FTUMP), four had oncocytic adenoma, 25 had follicular adenoma, four had thyroid cysts, 89 had papillary thyroid carcinoma, one had invasive encapsulated follicular variant of PTC (IEFVPTC), three had follicular thyroid carcinoma (FTC), one had oncocytic carcinoma, two had poorly-differentiated thyroid carcinoma, and nine had medullary thyroid carcinoma (MCT) (37). One patient was diagnosed with Gardener syndrome and columnar subtype of PTC, two with DICER1 and thyroid follicular nodular disease, and one with Cowden syndrome and oncocytic carcinoma. Nine patients received radiotherapy of central nervous system, CNS (ALL) or chest (Hodgkin Lymphoma, Neuroblastoma, Wilms tumor) at a mean of 8.8 (range 2-14) years before PTC confirmation.

Ninety patients out of this group (34.9%), all with confirmed PTC, with a mean age of 13.8 years (age range 6-18 years, 69 [76.7%] girls) were further evaluated and presented in the study.

Patients with PTC were divided into four groups depending on the presence (+) or absence (-) of autoimmune thyroiditis (AIT) and lymph node metastasis (LNM): the PTC AIT (+) LNM (+) group included 26 patients with a mean age of 13.8 years (range 7-18 years, 84.6% girls); the PTC AIT (+) LNM (-) group included 18 patients with a mean age of 15.3 years (range 8.8-18 years, 94.4% girls); the PTC AIT (-) LNM (+) group included 35 patients with a mean age of 12.4 years (range 6-18 years, 60% girls); and the PTC AIT (-) LNM (-) group included 11 patients with a mean age of 13.6 years (range 6-18 years, 81.8% girls) (**Tables 1**, **2**).

**Table 1:** Auxological, ultrasound, cytologic and histopathologic characterisation of patients diagnosed with PTC (n=90).

PTC (n=90) features	PTC (+) AIT (+) n=44 (48.9%)		PTC (+) AIT (-) n=46 (51.1%)		p
	LNM (+)n=26 (59.1%)	LNM (-)n=18 (40.9%)	LNM (+)n=35 (76.1%)	LNM(-)n=11 (23.9%)	
**Age mean [range]**	13.8 [7-18]	15.3 [8.8-18]*	12.4 [6-18]*	13.6 [6-18]	0.01
**Gender, Female %**	84.6	94.4	60	81.8	0.71
**Presentation %** **Goiter** **Ultrasound** **Lymphadenopathy**	50% 50%	16.7% 83.3%	62.8% 31.4% 5.8%	45.4% 54.6%	0.42 0.44
**Risk groups**	1/26 - 7 yrs after Rtx (ALL)	Gardener s.	3/35 - 2,7,11 yrs after Rtx (ALL) 1/35- 9 yrs after Rtx (Wilms Tu.) 1/35- 3 yrs after Rtx (Hodgkin D.)	2/11- 9,13 yrs after Rtx (ALL) 1/11- 14 yrs after Rtx (NBL)	–
**Familial PTC**	2/26		1/35		–
**Recurrence**	1/26		4/35		–
**Single nodule seen on thyroid US %**	50	88.9	57.1	81.8	0.35
**Lesion seen on thyroid US %**	50	11.1	42.9	18.2	0.45
**The largest dimension of nodule/lesion on US in mm** **mean [range; SD]**	**21.2** [4-52; 14.3]*	**10.4** [6-16; 3.9]*,#	**23.9** [5-60; 16.6]*,#	13.9 [4-60; 9.0]	0.01
**PTC** **unilateral %** **multifocal and bilateral%**	19.2 80.8	61.1 38.9	20 80	72.7 27.3	0.72 0.67
**EU-TIRADS mean%**	5 [100%]	5 [100%]	5 [100%]	5 [100%]	0.68
**Pathological (round, hypo-or hyperechoic and hypervascular) lymph nodes seen on US (% of patients)**	91%	–	88%	–	0.78
**Largest dimension of the LNM in mm mean [SD]**	9.4 [8.5]	–	6.9 [8.5]		0.71
**T%** **1a** **1b** **2** **3** **4**	26.9 26.9 30.8 15.4 -	83.3* 16.7 - - -	22.8* 34.4 20 20 2.8	36.4 45.4 18.2 - -	0.01 0.32 0.42 0.41 -
**N%** **1a** **1b**	61.5* 38.5*	- -	25.7* 74.3*	- -	0.01 0.01
**M%** **1** **0**	19.2 80.8	- -	20 80	- -	0.51 0.42
**Bethesda** **mean [range]**	3.6 [2-5]	4.9 [2-6]	5.1 [3-6]	4.5 [2-6]	0.54
**LI Ki67** **mean [range]**	10.6 [3-20]	11.2 [10-15]	8.9 [3-20]	9.5 [8-20]	0.45
**PTC subtypes,** **n [%]:** **Classic(C)** **Follicular (F)** **C,F,S** **C,S** **C,F** **S,F** **D.S.** **Columnar** **C,F,S,T,G,C-M** **C,F,S,C-M** **C,F,S,O,C-M** **F,S, D.S** **S, anaplastic** **Invasive encapsulated follicular variant papillary thyroid carcinoma**	11 [42.3] 6 [23.1] 4 [15.4] 2 [7.7] 2 [7.7] 1 [3.85]	11 [61.1] 1 [5.56] 1 [5.56] 2 [11.1] 1 [5.56] 1 [5.56] 1 [5.56]	17 [48.6] 3 [8.6] 4 [11.4] 4 [11.4] 1 [2.85] 1 [2.85] 1 [2.85] 1 [2.85] 1 [2.85] 1 [2.85] 1 [2.85]	6 [54.5] 4 [36.4] 1 [9.1]	0.43 0.45 0.51 0.54 0.53 0.64 0.42 - - - - - - -

PTC, papillary thyroid carcinoma; AIT, autoimmune thyroiditis; C, classic; F, follicular; S, solid; O, oxyphylic; D.S., diffuse sclerosing; C-M, cribriform-morular; T, tall cell; G, glomerular; PTC, subtypes; LNM, lymph node metastasis (+) present, (-) absent, US-ultrasonography, Rtx-radiotherapy, NBL-neuroblastoma, D-disease, S-syndrome, ALL-acute lymphoblastic leucemia. Postoperative staging based on the tumour (T), nodes (N) and metastases (M)-TNM system proposed by the American Joint Committee on Cancer (54). Data are expressed as mean [range, SD] or %.*,#-p<0.05. Bold values represent significant differences between the data.

**Table 2:** Thyroid status in patients with papillary thyroid carcinoma (PTC, n=90) with or without coincidence with autoimmune thyroiditis [AIT(+)/(-)] and LMN(+)/LNM(-) compared with AIT (+) PTC (-) control group (n=135).

	PTC (+) GROUP n=90				AIT (+) PTC (-) CONTROL GROUP n=135		
Patients characteristics in PTC (+) group/ Ultrasound pattern in control group	PTC AIT(+) LNM (+)	PTC AIT(+) LNM (-)	PTC AIT(-) LNM (+)	PTC AIT(-) LNM (-)	Diffuse thyroiditis	Diffuse thyroiditis with irregular background	Micronodulations (pseudonodules)
n	26	18	35	11	61	52	22
TSH mean [SD] uIU/ml (n:0.4-4.0)	4.2 a [6.7]	4.1 b [6.1]	2.4 # [1.2]	3.01 [1.5]	62.2 a,b,#,* [133.6]	5.6 * [9.2]	16.8 [53.4]
fT3 mean [SD] pmol/l (n:3.6-6.8)	7.9 [6.9]	5.7 [3.2]	5.5 [2.7]	5.5 [1.1]	9.9 [10.3]	11.9 [8.9]	8.3 [9.1]
fT4 mean [SD] pmol/l (n: 10-25)	13.9 [13.2]	9.8 [8.8]	12.1 [5.7]	11.6 [6.2]	16.2 [17.9]	20.9 [17.1]	13.7 [10.8]
T%	11.5	–	–	–	12.7	27.8	4.8
E%	80.8	83.3	88.6	90.9	17.5	35.2	52.4
H%	7.7	16.7	11.4	9.1	69.8	37	42.8
TPOAb mean [SD] IU/ml (n<30)	772.9 a [1838]	1398.9 # [2576]	–	–	5641.7 a,#,*,b [3500]	1849 * [2030]	1712.4 b [1760]
TgAb mean [SD] U/ml (n<20)	1358.2 a,c,d [1598.2]	637.8 a,# [1781.6]	–	–	1474.2 #,*,b [2642.1]	347.1 *,c [947.1]	792.3 b,d [1755.6]
TRAb mean [SD] U/ml (n<1)	1.7 [1.3]	1.7 [1.9]	–	–	3.8 [6.05]	5.4 [7.2]	2.8 [3.0]

T, Hyperthyroid; E, Euthyroid; H, Hypothyroid. Data are expressed as mean [SD] or as %. *,#,a,b,c,d- statistically significant differences between the means in the groups (*,#,a,b,c,d-represents differences between two groups), p=0.01. Mean TSH and mean TPOAb levels were significantly higher in control group with diffuse thyroiditis than in other groups (bold). Mean TgAb was significantly higher in patients with PTC AIT (+) LNM (+) and control group with diffuse thyroiditis than in other groups (bold). Bold values represent significant differences between the data.

The control group included 135 age- and sex-matched paediatric patients with AIT and without PTC [AIT (+) PTC(-)] (**Table 2**).

Patients with PTC were also divided into three age groups: prepubertal (<10 years of age), pubertal (age range 11-14 years), and late pubertal (age range 15-18 years, all female patients had regular menses) (**Table 3**). Puberty was assessed by researchers DJ and MW, based on the Tanner scale. Prepubertal: Thelarche/Breast I, Pubarche/Pubic hair-I, Axillarche-I, testes volume < 4 ml each.

**Table 3:** Auxological, ultrasound, cytologic and histopathologic characterisation of patients diagnosed with PTC (n=90) presented in age groups.

Parameter	Age groups			p
Age groups [years]	<10	11-14	15-18	
Mean age [years]	8.2	12.9	16.8	0.62
n	19	35	36	0.71
Females%	52.6	80	86.1	0.11
Presentation:				
Goiter% Ultrasound% Lymphadenopathy%	63.1 26.3 10.6	54.3 45.7 -	30.5 69.5 -	0.22 0.32
PTC mm mean [SD]	25.4* [15.2]	20.2# [14.8]	15.6*,# [11.6]	0.01
ETE%	68.4*	45.7	13.9*	0.01
AI%	63.1*	89.5#	27.7*,#	0.02
≥2 subtypes%	57.9*	34.3	16.7*	0.02
1 subtype%	42.1*	65.7	83.3*	0.02
PTC Subtypes (n)				
classic follicular solid oxyphilic diffuse sclerosing cribriform-morular tall cell columnar glomerular anaplastic+solid	14 11 10 1 2 1 - - 1 1	28 17 5 - 1 1 1 - - -	25 12 5 - - - - 1 - -	0.21 0.22 0.12 - - - - - - -
LI KI67 mean [SD]	11.25 [7.04]	9.5 [5.8]	9.7 [5.8]	0.45
Fibrosis%	57.9	54.3	44.4	0.55
Psammoma bodies%	57.9	54.3	33.3	0.65
LNM%	78.9*	71.4	58.3*	0.04
Recurrence%	10.5	8.6	–	0.25
AIT%	31.6	48.6	61.1	0.35
TSH mean [SD] mIU/ml	4.9 [7.8]	3.3 [4.1]	2.5 [2.4]	0.54
TPOAb mean [SD] IU/ml	149.7 [268.4]	440.3 [1557.3]	880.0 [1953.0]	0.55
TgAb mean [SD] U/ml	272.2 [426.1]	375.2 [1430]	533.0 [1519]	0.21
131 I therapy%	94.7	82.8	86.1	0.25

PTC, papillary thyroid carcinoma; AIT, autoimmune thyroiditis; C,classic; F,follicular; S,solid; O,oxyphylic; D.S.,diffuse sclerosing; C,M,cribriform,morular; T,tall cell; G,glomerular; PTC subtypes; LNM,lymph node metastasis; ETE,extrathyroidal extension, AI-angioinvasion. Data are expressed as mean [SD] or as %.*,#-p<0.05. Bold values represent significant differences between the data.

**Figure 1:**
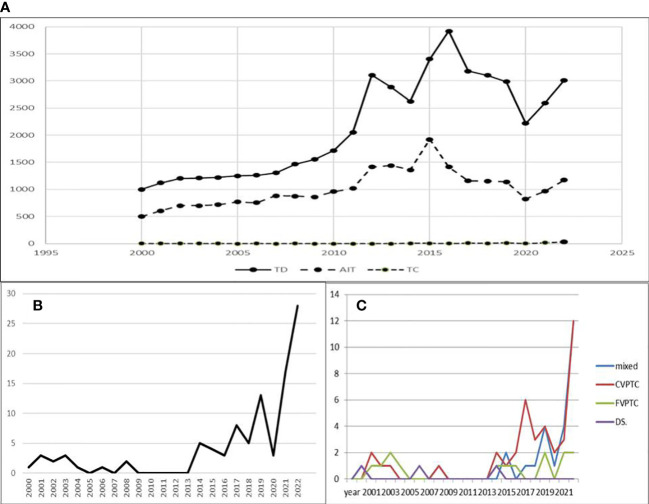
**(A)** The number of appointments in the Thyroid Clinic per year observed since 2000. COVID-19 pandemic temporarily decreased the number of appointments. **(B)** Increase in PTC cases per year since 2000. **(C)** Increase in classic and mixed subtypes in last three years. Legend: TD-thyroid disorders, AIT-autoimmune thyroiditis, TC-thyroid cancer, mixed-mixed subtypes of PTC, CVPTC-classic subtype of PTC, FVPTC-follicular subtype of PTC, DS-diffuse sclerosing subtype of PTC.

Since 2000, we have observed an increase in patient visits due to thyroid disorders and AIT (**Figure 1A**). Patients with AIT represent ~1/3 of visits due to thyroid disorders (**Figure 1A**). This percentage has not significantly increased since the beginning of our observation because of the overall increase in the number of patients referred to our centre annually for prophylactic assessment of thyroid function (**Figure 1A**). However, from 2000 to 2022, we observed an increased incidence of thyroid cancer in 105 patients, including 90 patients with PTC (**Figure 1B**).

PTC in combination with AIT was found in 48.9% (44/90) of patients (**Tables 1**, **2**). Patients with PTC AIT(+) were older than those with PTC AIT (-). Both groups were further subdivided according to the presence or absence of lymph node metastasis (LNM). The youngest patients were in the PTC AIT (-) LNM (+) group (**Table 1**). The highest percentage of female patients was found in PTC AIT (+) LNM (-) group, and the lowest was found in PTC AIT(-) LNM (+) group: 94.4 vs. 60% (**Table 1**). The youngest patients in PTC AIT(-) LNM(+) group presented more often with goiter or lymphadenopathy than patients from other groups (**Table 1**).

Out of 9 patients who developed PTC after radiotherapy of the CNS (due to acute common leukaemia, ALL) or chest (Wilm’s Tumour, Hodgkin Disease, Neuroblastoma), only one patient with ALL (without bone marrow transplantation) developed AIT.

In 34/90 (37.8%), there were thyroid disorders in the first-degree relatives, including PTC in mothers of three patients and one family with Gardener syndrome.

### Ultrasound variables

**Figure 2:**
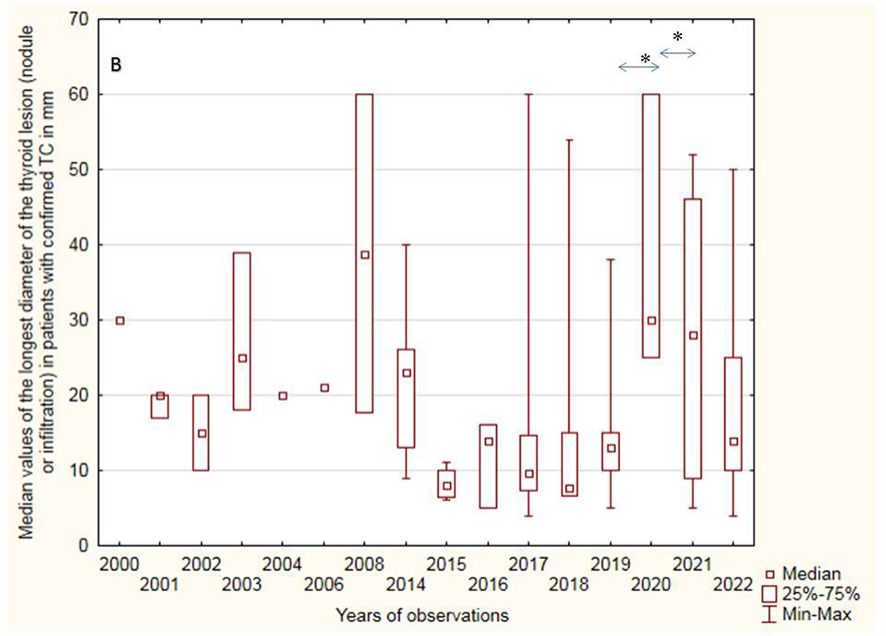
Median values of the longest diameter of the thyroid lesion (nodule or infiltration) in patients with confirmed PTC (mm). Significant increase of PTC diameter in COVID-19 pandemic compared to year before and after pandemic (*p=0.01).

During the 22 years of our ultrasound observations, the length of nodules detected on US decreased significantly, as we actively surveilled patients with thyroid disorders, as described previously; however, after the COVID-19 pandemic, we observed a significant increase in the diameter of nodules and observed patients with more advanced disease (**Figure 2**) (24).

**Figure 3:**
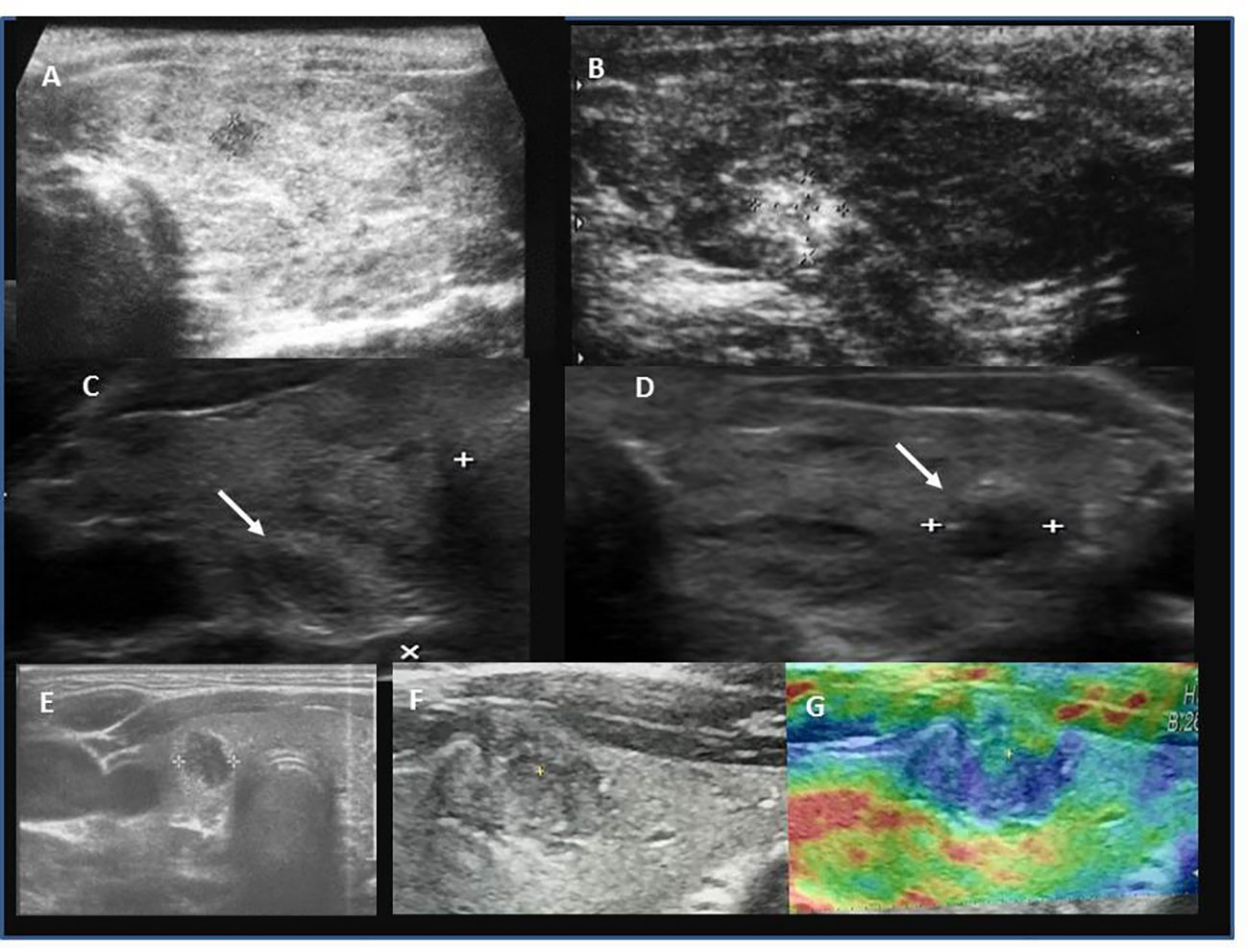
Ultrasound presentation of thyroid nodules confirmed as PTC in patients AIT (+). **(A)** female, 17.5 years, multifocal (M), bilateral (B), PTC 6.6 mm, classic, follicular; **(B)** female, 17.5 years, unilateral (U), PTC 9.8 mm, classic; **(C, D)** female, 18 years old, M,B, PTC 5 mm max lesion (white arrows), classic; **(E)** female, 13 years old, U, PTC 9 mm, classic, follicular, solid; **(F, G)** female, 15 years old, M,B, PTC 12 mm, classic, follicular, solid, G-elastography presented that malignant lesion was stiffer (blue colour) than surrounding thyroid tissue (green, yellow, red colour).

**Figure 4:**
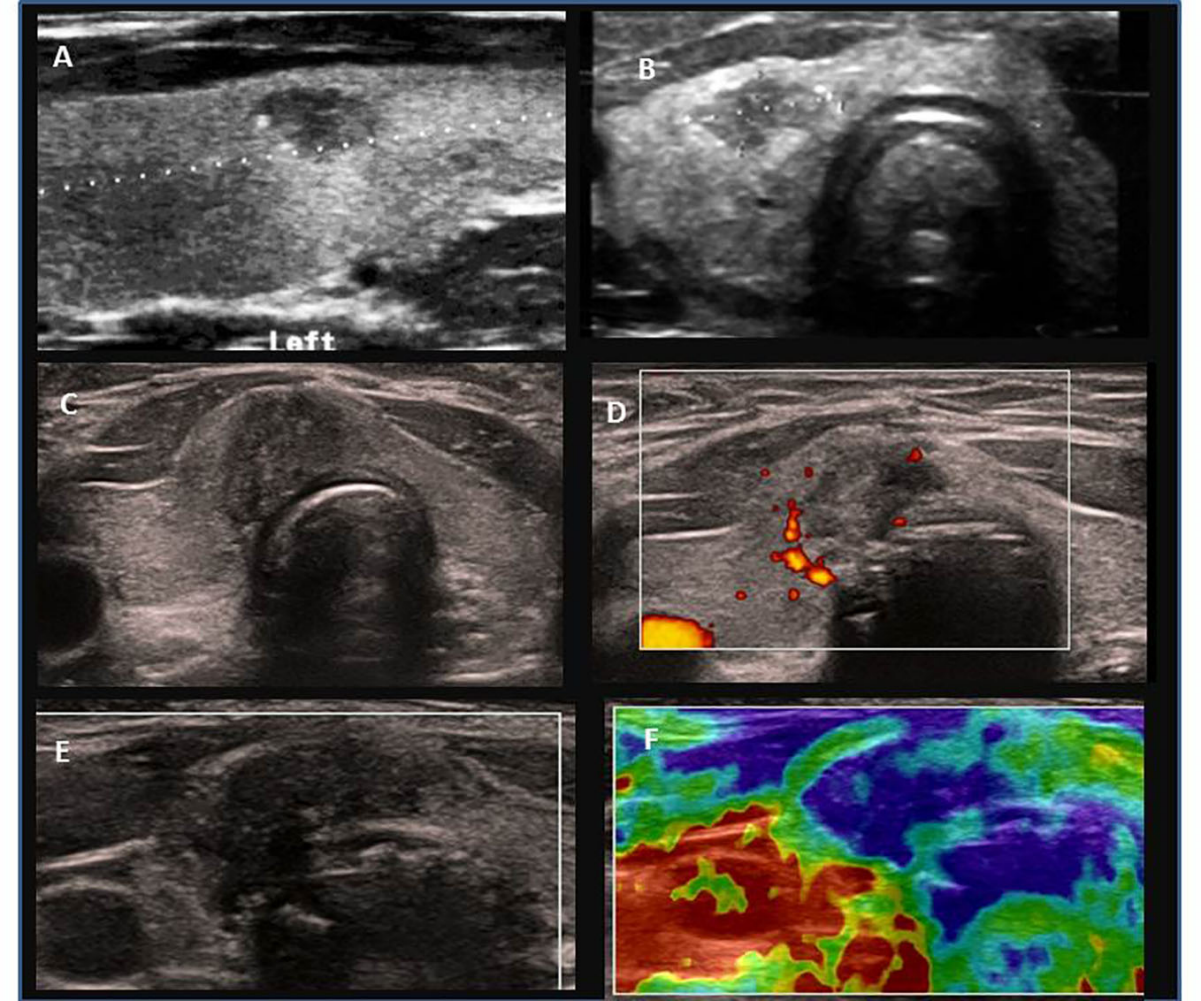
Ultrasound presentation of thyroid nodules confirmed as PTC in patients AIT (-). **(A)** female, 11.9 years old, multifocal (M), bilateral (B), PTC 7.6 mm, classic; **(B)** male, 15 years, M, B, PTC 9.5 mm, classic; **(C–F)** female, 14 years old, M,B, PTC 20 mm, classic, follicular, solid; **(D)** increased peripheral vascularization, **(F)** elastography presented that malignant lesion was stiffer (blue colour) than surrounding thyroid tissue (green, yellow, red colour).

**Figure 5:**
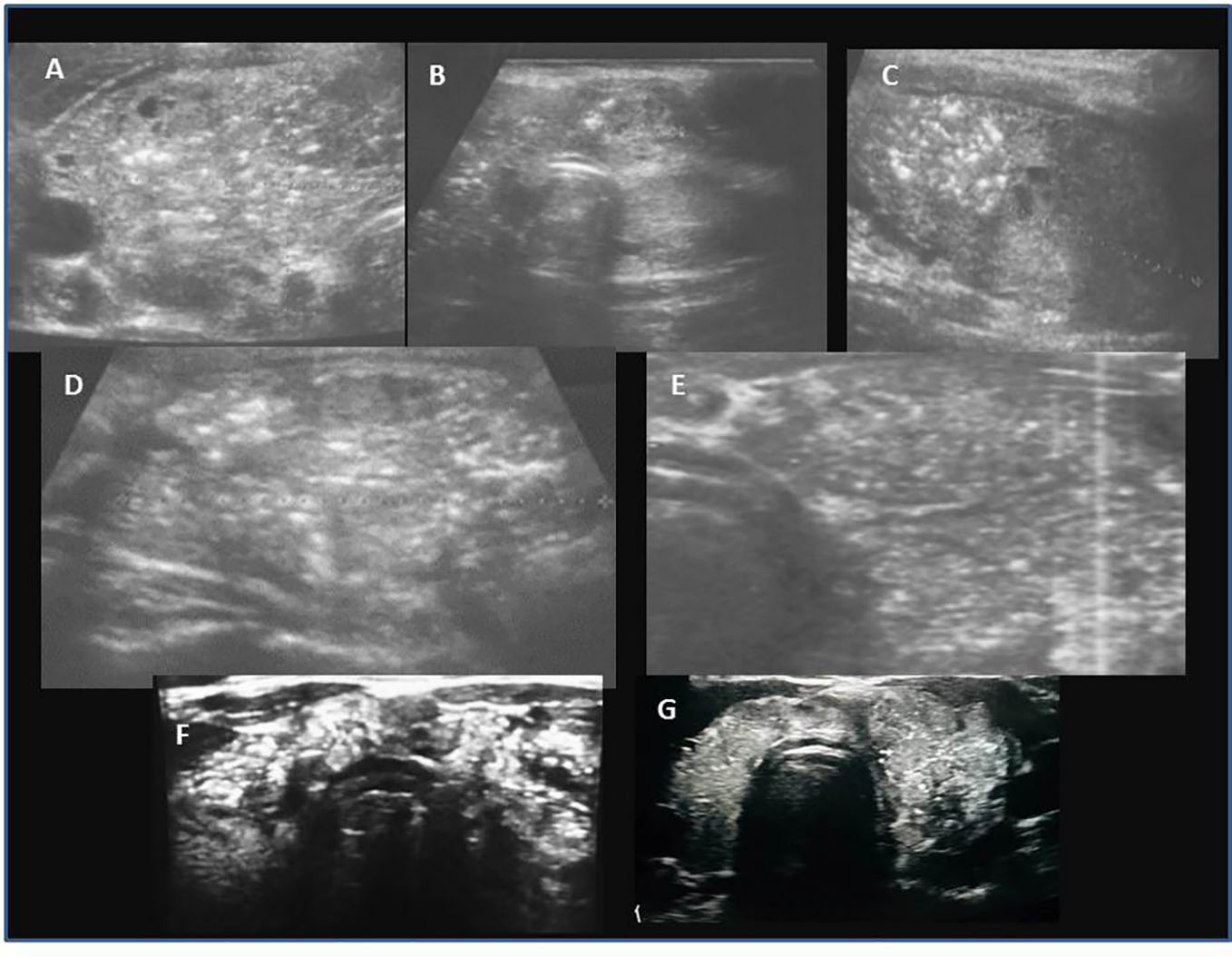
PTC in three patients presenting with thyrotoxicosis. **(A–C)** female, 12 years old, multifocal and bilateral lesion, classic PTC; **(D, E)** female, 18 years old, multifocal and bilateral lesion, classic PTC; **(F, G)**-male, 12 years old, multifocal and bilateral lesion, classic PTC.

Among patients with PTC, hypoechogenic malignant nodules predominated over other ultrasound patterns (**Figures 3**, **4**). In three patients with thyrotoxicosis PTC presented as lesions (**Figure 5**).

Ultrasound assessment revealed a single thyroid nodule in ~50% patients LNM(+) and in over 80% patients LNM (-) (**Table 1**). In 39 patients, lesions were unilaterally localised in the right lobe, in 21 patients in the left lobe, and in 30 patients lesions were located bilaterally. In 85% of patients with nodules, the single nodule was localised in the lower pole, between the isthmus and the lobe (**Figures 3**, **4**). However, histopathological analyses revealed that unilateral nodules were more often found in PTC AIT (-) and LNM (-), and multifocality was found more frequently in LNM (+), independent of AIT (**Table 1**). The positive predictive value (PPV) that in a case of a single nodule seen on US PTC was unilateral was 53.3%, and the negative predictive value (NPV) was 46.7%. The PPV that in a case of a single nodule seen on US there are no LNM was 40% and the NPV was 60%. This observation explains why pediatric patients are subjected to total thyroidectomy with central and, if necessary, lateral LNM dissection.

The largest nodule size was seen in patients with PTC AIT (-) LNM (+) and the smallest in PTC AIT (+) LNM (-) (**Table 1**).

The assessment of thyroid nodules or lesions with the use of EU-TIRADS PL scale showed 100% concordance with histopathological assessment (**Table 1**).

**Figure 6:**
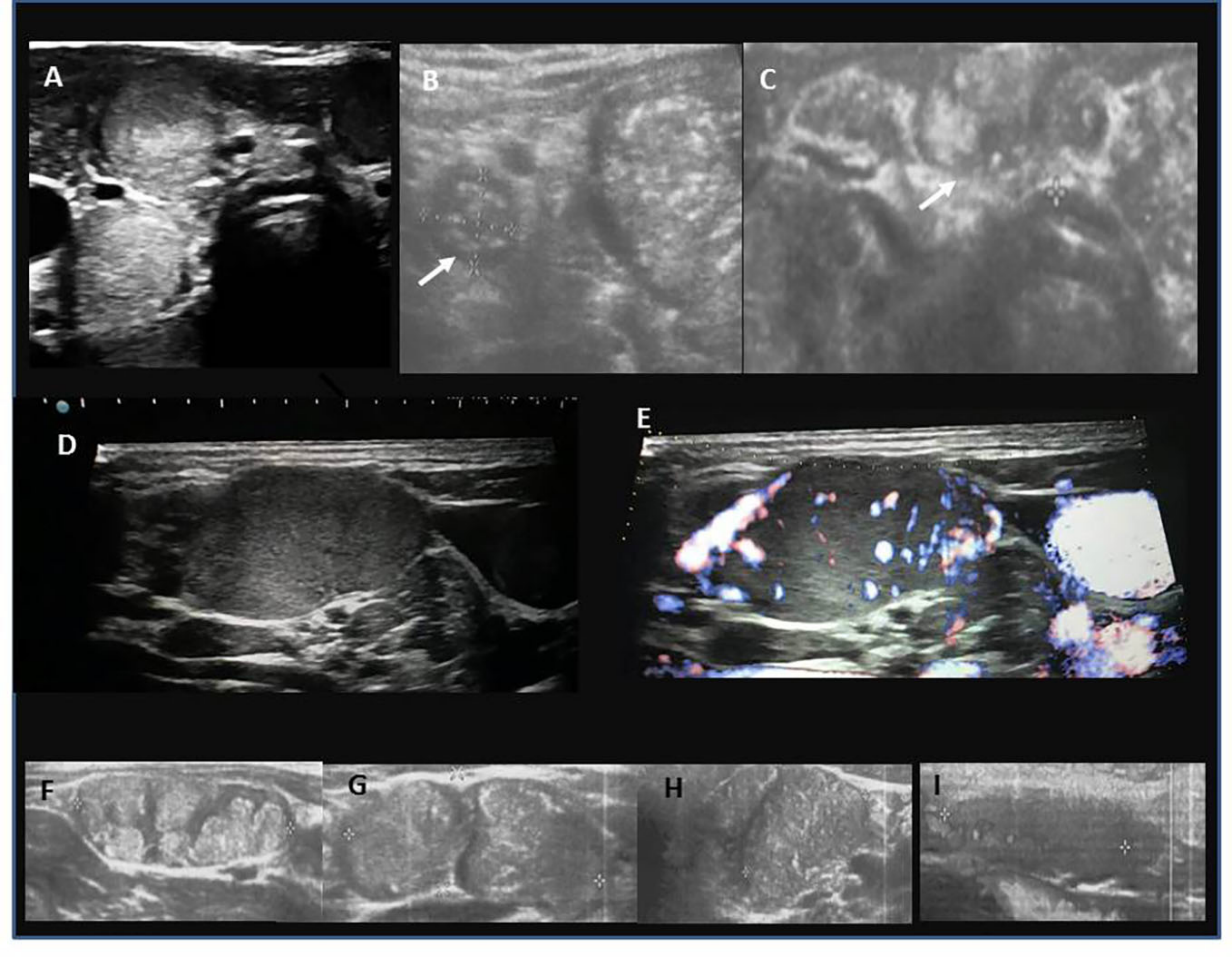
Ultrasound presentation of metastatic lymph nodes (LNM; central, C LNM; lateral, L LNM). **(A)** round C LNM with microcalcifications; **(B)** round L LNM with microcalcifications (white arrow), the echostructure of LNM is similar to the echostructure of the thyroid tissue with PTC lesion next to it; **(C)** oval C LNM with microcalcifications (white arrow); **(D)** solid, L LNM; **(E)** increased chaotic vascularization in L LNM; **(F)** nodular, irregular structure of L LNM; **(G, H)** microcalcifications in L LNM; **(I)** hypoechogenic L LMN without hilum.

Pathological lymph nodes seen on ultrasound (hypo- or hyperechoic and oval or round, with nodularity inside and hypervascular) that were later confirmed to contain metastasis were observed and described in 91% of patients with AIT(+) LNM (+) and 88% AIT (-) LNM (+) (**Figure 6**; **Table 1**). The calculated PPV for ultrasound suspicion of LNM was 94.9%; the NPV, sensitivity, and specificity were 81.8%, 90.3%, and 90%, respectively.

### Cytopathology

The Bethesda score was the highest in the AIT(-) LNM (+) subgroup (**Table 1**).

### Histopathology

In patients from AIT (-) LNM (-) group the percentage of mixed PTC subtypes was lower than that in other groups (**Table 1**). No difference was found in the LI Ki67 index between groups (**Table 1**). Analysis of histological subtypes in the following years revealed an increase in classic and mixed subtypes (classic/follicular/solid/cribriform/morular/tall cell) (**Figure 1C**).

### TNM evaluation

Significantly more patients with T1a stage were observed in AIT(+) LNM(-) than in AIT (-) LNM(+) group. Stage N1a was observed more often in AIT (+) LNM(+) than in AIT (-) LNM (+), in contrast to N1b stage. The percentage of M1 was similar in both groups (**Table 1**).

### Thyroid status

Thyroid status in patients with PTC with or without autoimmune thyroiditis [AIT(+)/(-)] compared with the most common variants of AIT in control group [AIT (+) PTC(-)] is presented in **Table 2**. In patients with PTC LNM(+), independently on AIT, TSH was significantly lower than in patients with diffuse thyroiditis; in the former groups, 11.5% patients presented with thyrotoxicosis, over 80% were euthyroid, and 7.7% and 11.4% were hypothyroid. In the latter group, almost 70% were hypothyroid. The majority of patients with PTC AIT (-) was euthyroid. The highest TSH was observed in diffuse thyroiditis, and the lowest was observed in PTC AIT(-) LNM(+) group. The mean TPOAb level was higher in patients with diffuse thyroiditis than in the other groups. Mean TgAb level was higher in AIT with diffuse thyroiditis and in PTC AIT (+) LNM(+) groups than in AIT with irregular background or micronodulations and in PTC AIT(+) LNM(-) groups. Coincidence of PTC with thyrotoxicosis was found in three patients in AIT (+) LNM(+) group.

### Age groups

Evaluation of PTC variables by age groups (**Table 3**) revealed that there were more boys in the youngest group of patients (< 10 years of age) than in both the older age groups. The F:M ratio was close to equal in the prepubertal group, and 4:1 in pubertal and late pubertal groups (**Table 3**). Patients in the youngest group more often presented with goitre, whereas a single nodule was found incidentally on ultrasound evaluation more often in the oldest patients (**Table 3**). The mean dimension of PTC was significantly larger, and the percentage of ETE, AI, mixed subtypes, fibrosis, psammoma bodies, and LNM was higher in the youngest patients than in the oldest age group (15-18 years of age) (**Table 3**). We found significant negative correlations between younger age and size of the primary tumour (r:-0.3, p=0.01), LNM (r:-0.26, p=0.02), diameter of the largest LNM (r:-0.31, p=0.01), ETE (r:-0.3, p=0.01), AI (r:-0.25, p=0.02), labeling index of a proliferation marker Ki67 (LI Ki67) (r:-0.26, p=0.03), psammoma bodies (r:-0.31, p=0.01), fibrosis (r:-0.32, p=0.02) and Bethesda score (r:-0.3, p=0.04). The percentage of recurrence was also higher in the youngest patients, but not significantly.

There were no differences in TPOAb, TgAb, and TSH levels between the age subgroups, but the percentage of AIT and the mean TPOAb level were higher in the oldest group. TgAb levels were higher than those of TPOAb in the youngest patients (**Table 3**). Histopathological analysis revealed that the solid subtype was observed more often in prepubertal children and diffuse sclerosing in children below 14 years of age, whereas the classic subtype dominated in late pubertal (15-18 years of age) (**Table 3**). Except for 1 patient with a PTC diameter of 6.7 mm, all prepubertal patients received ^131^I therapy. In the other age groups, 6/35 and 5/37 children did not receive ^131^I therapy (**Table 3**).

### Associations

Univariate and multivariate analyses revealed that LNM was associated with PTC diameter and fT4 level, whereas extrathyroidal extension with age, and angioinvasion with PTC diameter and age (**Tables 4**, **5**). The correlations between age and fibrosis, and the presence of psammoma bodies in malignant tissues were close to significant (**Table 5**). We did not observe an association between TSH levels and the presence of autoimmunity and PTC variables (**Tables 4**, **5**).

**Table 4:** Univariate and multivariate logistic regression analysis of variables influencing LNM, ETE and AI.

	LNM-	LNM+	Univariate		Multivariate	
			OR (CI)	p	OR (CI)	p
Female sex	24 (83)	44 (71)	0.51 (0.16-1.57)	0.24	–	–
Age (years)	14.7 (6-18)	13 (6-18)	0.84 (0.73-0.98)	0.028	0.90 (0.76-1.06)	0.21
Family history	13 (45)	21 (34)	0.63 (0.25-1.57)	0.32	–	–
PTC (mm)	11 (4-32)	20 (4-60)	1.09 (1.02-1.15)	0.004	1.09 (1.03-1.16)	0.0026
AIT	18 (62)	27 (43)	0.47 (0.18-1.18)	0.11	–	–
TSH uIU/ml	2.6 (0.05-26)	2.16 (0-36)	0.98 (0.89-1.07)	0.60	–	–
fT4 pmol/l	12.9 (0.5-17.8)	14.2 (0.9-70.1)	1.07 (0.99-1.14)	0.08	1.09 (1.01-1.18)	0.032
	ETE-	ETE+	Univariate		Multivariate	
			OR (CI)	p	OR (CI)	p
Female sex	40 (74)	28 (75)	1.09 (0.40-2.90)	0.86	–	–
Age (years)	14.6 (6-18)	12 (6-18)	0.83 (0.73-0.95)	0.009	0.86 (0.75-0.99)	0.044
Family history	23 (42)	11 (30)	0.57 (0.23-1.4)	0.22	–	–
PTC (mm)	13 (4-60)	19 (4-60)	1.03 (1.0-1.07)	0.038	1.02 (0.99-1.06)	0.13
AIT	30 (55)	15 (40)	0.54 (0.23-1.29)	0.16	–	–
TSH uIU/ml	2.4 (0.02-36)	1.9 (0-26)	0.99 (0.91-1.09)	0.93	–	–
fT4 pmol/l	13.1 (0.81-31.6)	13.9 (0.5-70.1)	1.01 (0.97-1.07)	0.45	–	–
	Angioinvasion (-)	Angioinvasion (+)	Univariate		Multivariate	
			OR (CI)	p	OR (CI)	p
Female sex	36 (76)	32 (73)	0.81 (0.31-2.13)	0.67	–	–
Age (years)	15 (6-18)	13 (6-18)	0.79 (0.68-0.92	0.02	0.83 (0.71-0.96)	0.016
Family history	19 (40)	15 (34)	0.76 (0.32-1.81)	0.53	–	–
PTC (mm)	11 (4-60)	18 (4-60)	1.05 (1.01-1.09)	0.006	1.04 (1.0-1.08)	0.033
AIT	27 (57)	18 (41)	0.51 (0.22-1.19)	0.12	–	–
TSH uIU/ml	2.16 (0.02-26)	2.38 (0-36)	1.02 (0.93-1.12)	0.65	–	–
fT4 pmol/l	13.1 (0.5-31.6)	13.9 (0.9-70.1)	1.01 (0.97-1.07)	0.47	–	–

LNM, lymph node metastasis; ETE, extrathyroidal extension; AIT, autoimmunity. Only characteristics with p<0.1 in univariate analysis were included in the multivariate analysis, as p<0.1 is considered as a trend, p<0.05 as significant.

**Table 5:** Univariate logistic regression analysis of variables influencing fibrosis, the appearance of psammoma bodies, unilaterality and multifocality of PTC.

	Without fibrosis		Fibrosis	Univariate		
				OR (CI)		p
Female sex	30 (71.4)		38 (77.5)	1.38 (0.53-3.61)		0.50
Age (years)	14.2 (6-18)		14 (6-18)	0.91 (0.80-1.03)		0.14
Family history	15 (36)		19 (39)	1.14 (0.48-2.70)		0.76
PTC (mm)	14 (4-60)		14 (4-60)	1.02 (0.99-1.05)		0.16
AIT	19 (45)		26 (53)	1.37 (0.59-3.16)		0.45
TSH uIU/ml	2.28 (0.02-26)		2.16 (0-36)	1.01 (0.93-1.11)		0.71
fT4 pmol/l	13.4 (0.5-31.6)		13.4 (0.81-70.1)	0.98 (0.93-1.03)		0.52
	No psammoma bodies		Psammoma bodies	Univariate		
				OR (CI)		p
Female sex	33 (71.7)		35 (77.8)	1.38 (0.52-3.61)		0.51
Age (years)	14.25 (6-18)		13 (6-17)	0.90 (0.79-1.02)		0.11
Family history	16 (35)		18 (40)	1.25 (0.53-2.96)		0.61
PTC (mm)	15 (4-60)		13 (4-60)	0.99 (0.97-1.02)		0.87
AIT	20 (43)		25 (55)	1.65 (0.70-3.76)		0.25
TSH uIU/ml	2.11 (0.02-9.66)		2.3 (0-36)	1.10 (0.95-1.29)		0.20
fT4 pmol/l	13.25 (0.97-31.6)		13.4 (0.5-70.1)	0.99 (0.95-1.04)		0.89
	Unifocal		Multifocal and bilateral	Univariate		
				OR (CI)		p
Female sex	20 (62.5)		48 (81)	2.69 (0.97-7.00)		0.55
Age (years)	14 (6-18)		14 (6-18)	0.98 (0.86-1.12)		0.79
Family history	15 (47)		19 (32)	0.53 (0.22-2.62)		0.17
PTC (mm)	12.6 (4-51)		16 (4-60)	1.04 (1.0-1.08)		0.4
AIT	17 (53)		28 (47)	0.80 (0.33-1.90)		0.61
TSH uIU/ml	2.5 (0.64-9.66)		2.1 (0-36)	1.02 (0.92-1.14)		0.67
fT4 pmol/l	13.25 (0.89-20.2)		13.4 (0.5-70.1)	1.0 (0.95-1.05)		0.95

LNM, lymph node metastasis; ETE, extrathyroidal extension; AIT, autoimmunity. As only characteristics with p<0.1 in univariate analysis could be included in the multivariate analysis, therefore multivariate analysis was not performed in these groups.

## Discussion

This cross-sectional retrospective study evaluated paediatric patients diagnosed with thyroid cancer at our centre since 2000. According to the WHO 2022 histologic classification of thyroid neoplasms, 96 cases of thyroid cancer included malignant follicular cell-derived neoplasms: three patients with FTC, one with IEFVPTC, 89 with PTC, one with oncocytic carcinoma of the thyroid, two with high-grade follicular-derived carcinomas (poorly differentiated TC), and nine with thyroid C-cell-derived carcinoma (MTC) (37). At present, we observed an increase only in PTC, whereas FTC and MTC rates are stably low, as observed in other centres (36).

Ultrasonographic analysis of the thyroid gland provides useful diagnostic information. Among patients with PTC, hypoechogenic malignant nodules predominated over other ultrasound patterns and single nodules that were often localised between the isthmus and the lower part of the lobe. Whereas typically in AIT or AITD ultrasound imaging reveals a hypoechogenic thyroid background due to more or less advanced lymphocytic infiltration that can be diffuse or focal, in our PTC cohort, we observed that on ultrasound imaging, the inflammatory process in the majority of patients with PTC was present, but not very advanced (18, 36, 51, 55). Our observations revealed that prepubertal children presented with more advanced disease than pubertal children and adolescents did. We found significant negative correlations between younger age and size of primary tumour, Bethesda score, lymph node metastasis (LNM), diameter of the largest LNM, ETE, AI, LI Ki67, psammoma bodies and fibrosis.

In relation to autoimmune status, the percentage of AIT was increasing with age; AIT was present only in 1/3 of prepubertal, close to 50% in pubertal, and over 60% in adolescent patients. This observation is striking because over 70% of young prepubertal children referred to medical attention presented with tumour on the neck (goitre in 63.1% or lymphadenopathy in 10.6%), in contrast to adolescents in whom PTC was detected incidentally in ~60% during active ultrasound surveillance of autoimmune thyroiditis.

Our observations are in line with two large studies on PTC in young patients. Demidchik et al. found that recurrent nodal disease and pulmonary metastasis were associated with younger age in children exposed to ionizing radiation after the Chernobyl accident in 1986 (56).

Thiesmeyer et al., in the largest study of paediatric PTC so far, also reported that prepubertal children presenting with the most extensive disease were most likely to have lymph node metastasis, extrathyroidal extension, nodal, and distant metastases at the time of diagnosis, even in the setting of small-diameter PTC (47). Prepubertal age compared to adolescent age is an independent predictor of nodal and distant metastatic disease (47).

He et al.in a retrospective study of 227 paediatric patients with PTC presented that 14 years is the best age cutoff to differentiate prepubertal from pubertal PTC, as it was one of the independent risk factors for progressive disease, suggesting that paediatric patients with PTC should not be considered as a single population (48). According to the ATA 2022 guidelines, a cutoff age of 18 years may be considered arbitrary, as the behaviour, natural history, and characteristics of PTC do not suddenly change at this age (36). Patients in the age group of 16–25 years may either have PTC that behaves as ‘typical’ childhood PTC or may have a more ‘adult’-like behaviour (36). Other studies have also implemented a cutoff for prepubertal PTC of 10 to 15 years (57–59). In our study, we accepted a cutoff of 10 years based on the physical examination of all prepubertal patients and previous reports by Liu et al. and Thiesmeyer et al. (47, 60).

The overall survival of our patients was excellent and mortality was null; however, we observed nodal recurrence in 5/90 children who were subsequently subjected to lateral lymphadenectomy. Recurrence was observed in prepubertal (<10 years) and pubertal children (<14 years of age). Studies have shown that the recurrence rate of PTC in children can reach 14–47% (60–62). In our study, the recurrence rate was 5.5%, which is lower than that in previous reports, probably related to the fact that 100% of the patients in our group underwent total thyroidectomy and 94.5% cervical neck dissection. Studies suggest that most cases recur within 5 years of diagnosis, but a few cases recur after 10 years or more, suggesting that some cases still have the possibility of future recurrence (63). Liu et al. found a greater rate of recurrence in children than in adolescents, and age was an independent variable predictive of recurrence (60). Therefore, for younger patients, the scope of surgical options should be more aggressive, and more attention should be paid to follow-up (60).

Although the advancement and recurrence rates of PTC are higher in prepubertal patients than in some adolescents, disease-specific mortality is low. The mechanism of this discrepancy is probably the differences in genetic profiles between young and prepubertal children and the greater sodium iodide symporter expression, which is related to radiosensitivity (44, 48).

The percentage of boys was higher and the F:M ratio was close to equal in prepubertal and 4:1 in older patients. Regarding sex distribution in the literature, the frequency of prepubertal PTC is higher than that of pubertal and adolescent PTC among males (48, 60, 64). Further investigations are needed to determine whether this result was caused by the small sample size of boys or whether puberty-related endocrine changes could also explain these differences (65, 66). This observation could be partially explained by the increase in thyroid autoimmunity with age, as observed in this study. Autoimmune thyroiditis is more frequent in female patients and increases with age (9). Younger patients tend to present late with more advanced disease owing to low awareness of cancer risk than actively surveilled older patients (30).

The almost 50% of coincidence of AIT and PTC in our study is concordant with two-fold increase in thyroid cancer risk in AIT paediatric patients presented in recent studies (67, 68). Similar to other studies, although AIT is associated with an increased risk of thyroid cancer, we did not find an association between AIT and the thyroid cancer stage at diagnosis (67). We believe that this could also be the effect of age and puberty on the different biological spectra of PTC in children, as suggested by He et al. and Thiesemeyer et al. (47, 48). In most cases of PTC, thyroid function tests are within normal limits (9, 18). Exceptionally rare cases of PTC have been associated with hyperthyroidism (9, 18). TSH has been proposed as a mediator of the association between AIT and thyroid cancer in adults with thyroid nodules (50, 67). However, evidence for an association between TSH and cancer among children with thyroid nodules has been conflicting (18, 19, 23, 31, 51). In this study, similar to a report by Keefe et al., TSH concentration was not associated with thyroid cancer (67). It is likely that the association of AIT with thyroid cancer in children is not mediated by elevated TSH concentrations and may be related more directly to thyroid inflammation/destruction, as we found an association between LNM and fT4 in patients not treated with levothyroxine or antithyroid thiamazole (67). These results corroborate data from Paparodis et al., who reported that the form of AITD pathology (destructive with clinically overt hypothyroiditis vs. less-destructive with clinically compensated hypothyroiditis or euthyroid) may play a role in differentiated thyroid cancer risk (55). Patients with less destructive AITD have a higher risk of differentiated thyroid cancer than those with destructive AITD (55). These observations support the hypothesis that autoimmune thyroiditis may be a secondary event.

Studies suggest that similar molecular mechanisms may influence the early stages of oncogenesis and inflammation in the thyroid gland (69). In our study, we found a significant difference in TPOAb levels between patients with AIT and those with AIT who developed PTC. In relation to preoperatively assessed TgAb levels, we found higher TgAb levels than TPOAb levels in younger patients. Additionally, we found significant differences in TgAb levels between patients with AIT(+) LNM(+) compared with patients with AIT(+) LNM(-). Thyroglobulin (Tg) and thyroperoxidase (TPO) are the main target antigens for cellular and humoral immune reactions (69–71). As presented by Ehlers et al., the tumour-protecting feature of TPOAb might be explained by (a) complement-mediated cell death, which is anti-TPO antibody-dependent because TgAb antibodies do not fix complement, and (b) TPOAb antibody-dependent cell toxicity due to the exclusive binding of anti-TPO antibodies to their effector cells *via* Fc-gamma receptor 1 (CD64), which is known to be expressed on monocytes (70). In contrast, TgAb seems to be a risk factor for PTC (70). One reason for this effect could be the fact that TgAbs from PTC patients recognise different Tg epitopes than TgAbs from patients with autoimmune thyroid diseases and from patients with PTC with associated thyroiditis (70, 71). Whether PTC develops despite autoimmunity or due to inflammation and preexisting autoimmunity, and whether AIT develops because of cross-reacting antitumour immunity, needs further research in paediatric patients (70, 71).

Although diagnostic criteria for PTC are the same regardless of patient age, differences in histotypes occur between adult and pediatric populations (72). High-risk histologic subtypes of PTC are reported to occur in 15–37% of paediatric PTC, including 7–13% tall-cell variant, 7–16% diffuse sclerosing variant, 1–4% solid/trabecular variant, and 2–6% poorly differentiated carcinoma (72). These subtypes are more aggressive, carry an increased risk of recurrence and mortality, and are therefore classified as high-risk histologies, while classical and follicular subtypes of PTC have a more favourable prognosis and are therefore classified as low-risk histologies (72–76). Low-risk subtypes (classic and follicular subtypes of PTC) remained the most commonly encountered subtypes seen in our study in adolescents, apart from two cases of poorly differentiated PTC, whereas an increased percentage of mixed and more aggressive subtypes was observed in children ≤14 years of age. This was reflected in a more aggressive surgical approach in younger patients with concomitant ^131^I therapy and more nodal recurrences.

In adult patients, incidental detection of small, clinically unapparent PTC, which is possible due to the increased availability of thyroid sonography, does not necessarily decrease mortality rates or improve patient health outcomes (77). Interestingly, Cancer Statistics from 2022 reveal that in adult population in The United States, after decades of increase, thyroid cancer incidence rates are now declining in both men and women at a combined rate of 2.5% per year from 2014 to 2018, partly because of recent changes in clinical practice designed to reduce over-detection (4). These changes are supported by data from autopsy studies, which indicate that the occurrence of clinically relevant thyroid tumours has remained stable since 1970 and is generally similar in men and women, despite a 3-fold higher overall incidence rate in women (4). However, in the most recent analysis published in 2023, the incidence of thyroid cancer increased in most countries among individuals irrespective of age (78). Moreover, the incidence increased in populations aged <40 years in several countries including Poland (78).

Therefore, we think that in cases of paediatric populations characterised by more advanced disease, children at risk, with AIT, with positive family history of thyroid problems (37.8% in our study), after radiotherapy, with lymphadenopathy, benefit from earlier detection, prior to lymph node metastasis, as it enables less aggressive surgical approach and might exclude from ^131^I therapy. ATA 2022 recommends that future studies be conducted to evaluate the impact of limited surgery for paediatric PTC with respect to recurrence and remission rates, considering the potential side effects of aggressive therapies (36). It was suggested that, in paediatric patients with incidentally found very small thyroid carcinoma and non-aggressive histological features, hemithyroidectomy may be considered a therapeutic option (36). Based on the results of the present study, such an attitude could be considered in some adolescents with early detected PTC.

The limitation of our study was the retrospective and institutional nature of the study, but the main advantage was that all researchers in this study were involved in therapy and monitoring of described patients, and it comprehensively presents patient evaluation from preoperative to concomitant ^131^I therapy, and includes histopathological evaluation.

Additionally, the limitations of our study relate not only to the limited sample size but also to the lack of tumour genetic profile information. We were unable to correlate the molecular differences between prepubertal and pubertal PTC. Recently, a few studies have shown that younger patients have a higher prevalence of fusion oncogenes than the *BRAFV600E* point mutation, which is regarded as the main molecular pathogenetic cause of adult PTC (45, 46). This finding suggests that diverse genomic alterations may exist in children, adolescents, and young adults with PTC.

The strength of our study was that we presented the associations between PTC variables not only in relation to puberty, dividing patients based on their examination of prepubertal (<10 years of age), pubertal (11-14 years), and adolescent/late pubertal, but also in relation to autoimmunity, assessed prior to thyroid surgery, and confirmed histopathologically.

## Conclusions

1. The youngest patients (<10 years old) present more often with goitre and lymphadenopathy and less often with AIT than adolescents (15-18 years of age).
2. In paediatric patients with AIT, the natural course of PTC may be less aggressive than that in patients with PTC AIT (-), partially because of active ultrasound surveillance and detection of smaller nodules prior to extensive lymph node metastasis.
3. We suggest that pre-operative evaluation of paediatric patients with thyroid nodules could include the assessment of TSH, fT3, and fT4, as well as the evaluation of TPOAb, TgAb, and TRAb together with comprehensive neck ultrasonography (thyroid and whole neck with lymph nodes).

## Data availability statement

The raw data supporting the conclusions of this article will be made available by the authors, without undue reservation.

## Ethics statement

The studies involving human participants were reviewed and approved by The Bioethics Committee of the Jagiellonian University opinion number: 1072.6120.288.2021. Written informed consent to participate in this study was provided by the participants’ legal guardian/next of kin. Written informed consent was obtained from the minor(s)’ legal guardian/next of kin for the publication of any potentially identifiable images or data included in this article.

## Author contributions

Study design: DJ. Study conduct: DJ, AT, AK-W, MK, MW, WG. Data collection: DJ, MK, AT, AK-W, MW. Data analysis: DJ, MW, MC, MK. Data interpretation: DJ, MW, MK, MC, AT, AK-W. Drafting manuscript: DJ, MW. Revising manuscript content: DJ, MW, WG, JS. Approving final version of manuscript: DJ, MW, JS, WG. DJ takes responsibility for the integrity of the data analysis. All authors contributed to the article and approved the submitted version.
